# Plumbagin reduces human colon cancer cell survival by inducing cell cycle arrest and mitochondria-mediated apoptosis

**DOI:** 10.3892/ijo.2014.2592

**Published:** 2014-08-08

**Authors:** BINIL ELDHOSE, MIA GUNAWAN, MAHBUBUR RAHMAN, MUKALEL S. LATHA, VICENTE NOTARIO

**Affiliations:** 1 School of Biosciences, Mahatma Gandhi University, Kottayam, Kerala-686560, India; 2 Department of Radiation Medicine, Lombardi Comprehensive Cancer Center, Georgetown University Medical Center, Washington, DC 20057, USA

**Keywords:** Plumbagin, medicinal herbs, cell cycle, apoptosis, colorectal cancer

## Abstract

Despite increased use of early detection methods and more aggressive treatment strategies, the worldwide incidence of colorectal cancer is still on the rise. Consequently, it remains urgent to identify novel agents with enhanced efficacy in prevention and/or therapeutic protocols. Our studies focused on the use of Plumbagin, a natural phytochemical that showed promising results against other tumor types, to determine its effectiveness in blocking the proliferation and survival of colon cancer cells in experimental protocols mimicking the environment in primary tumors (attached culture conditions) and in circulating tumor cells (unattached conditions). Under both experimental settings, exposure of HCT116 cells to Plumbagin concentrations in the low micromolar range resulted in cell cycle arrest at the G1 phase, apoptosis via the mitochondrial cell death pathway, and increased production of reactive oxygen species. The cell cycle effects were more noticeable in attached cells, whereas the induction of cell death was more evident in unattached cells. These effects were consistent with the nature and the magnitude of the alterations induced by Plumbagin on the expression levels of a set of proteins known to play key roles in the regulation of cell cycle dynamics, apoptosis mechanisms and cell proliferation. In light of its previously reported lack of toxicity on normal colon cells and the striking anti-survival effect on colon cancer cells observed in our study, Plumbagin should be considered a promising drug for the treatment of colon cancer.

## Introduction

Colorectal cancer (CRC) is one of the leading causes of death in the world, and is the third most common cancer in the United States (1). Recent US data on CRC incidence are alarming, with an estimated 103,170 cases, including 51,690 deaths, in 2012 (2), despite the increased use of early detection techniques. Therefore, it is essential to develop more aggressive prevention strategies as well as novel agents for CRC treatment. In recent years, natural products have received great attention as potential agents for cancer prevention or therapy owing to their health benefits and appreciably reduced toxicity and side effects, the major known limitations of most current chemotherapeutic agents (3). Natural compounds that block or suppress the proliferation of tumor cells and/or induce apoptosis are deemed to have potential as antitumor agents (4). Plumbagin (5-hydroxy-2-methyl-1, 4-naphthoquinone) occurs naturally in the medicinal herb *Plumbago zeylinica*, which has been safely used for centuries in Indian Ayurvedic and Oriental medicine for the treatment of various ailments (5–9). Plumbagin has attracted a great deal of research interest due to its known pharmacological effects as an anti-bacterial, hypolipidaemic, anti-atherosclerotic, Leishmanicidal and anticancer compound (10–16). In addition Plumbagin has been shown to be a potent radiosensitizer (17–19). In the present study, we explored the possible anticancer activity of Plumbagin on HCT116 colon cancer cells by analyzing its effects on cell cycle regulation, the expression of apoptosis-related signaling molecules and the formation of reactive oxygen species (ROS). Our data showed that Plumbagin had a dual effect on HCT116 cells: it caused G1 phase arrest by downregulating the expression of cyclin B1, cyclin D1 and NF-κB, and simultaneously promoted apoptosis by upregulating effectors of the mitochondrial pathway and inducing ROS formation. The magnitude of the apoptotic effect of Plumbagin was greater when cells were kept in suspension, whereas cell cycle effects predominated when treatments were performed on attached cells. As it was previously reported that Plumbagin does not show any toxicity on normal colon cells (20), our data suggest that, on the basis of predominant killing of cancer cells, Plumbagin should be considered as a promising drug for the treatment for colon cancer.

## Materials and methods

### Reagents

Plumbagin and DMSO, with purity >97%, were purchased from Sigma-Aldrich (St. Louis, MO, USA). A 100 mM stock solution of Plumbagin was prepared in DMSO, stored as small aliquots at −20°C, and then diluted as needed into cell culture medium. Penicillin-streptomycin solution, RPMI-1640 medium and fetal bovine serum (FBS) were obtained from CellGro (Manassas, VA, USA). Antibodies against AKT, BIM, PARP1, NF-κB, cyclin B1, BCL2, cleaved PARP1, caspase 3, cleaved caspase 3, caspase 9 and cleaved caspase 9 were purchased from Cell Signaling Technology (Beverly, MA, USA). The antibody to cyclin D1 was obtained from Santa Cruz Biotechnology (Santa Cruz, CA, USA); the FAK and Src antibodies were from BD Bioscience (San Jose, CA, USA), and the antibodies against p53 and p21^WAF1/CIP1^ were obtained from Epitomics (Burlingame, CA, USA) and EMD Millipore (Billerica, MA, USA), respectively. Ethidium homodimer was obtained from Sigma-Aldrich.

### Cell culture

The human colon cancer cell line HCT116 was obtained from the American Type Culture Collection (Manassas, VA, USA). All experiments were performed within three passages of cells cultured in RPMI-1640 supplemented with 10% FBS and 1% of a solution of penicillin (100 U/ml) and streptomycin (100 mg/ml). Cultures were incubated at 37°C, in an air atmosphere with 5% CO_2_ and 85% humidity.

### Cytotoxicity assays

The sensitivity of HCT116 cells to Plumbagin was determined by using the CellTiter-Glo^®^ luminescent cell viability assay in its 96-well format (Promega, Madison, WI, USA). Cells (8×10^4^) were exposed to different concentrations (≤10 μM) of Plumbagin for 24 h. Cells treated with DMSO served as control. After Plumbagin treatment, the CellTiter-Glo reagent (200 μl) was added to the culture medium in each well to induce cell lysis. After 10 min at room temperature (RT), the luminescence was recorded in a Berthold Microlumat Plus LV 96V luminometer from the Genomics and Epigenomics Shared Resource of the Lombardi Comprehensive Cancer Center (LCCC). Percentage of residual cell viability was determined by the ratio luminescence of treated cells/luminescence of control cells.

### Flow cytometry analysis

For cell cycle analysis, cells were harvested 24 h after exposure to Plumbagin, washed once in phosphate-buffered saline (PBS), fixed in 75% ethanol, resuspended in PBS containing 20 μg/ml propidium iodide (EMD Millipore), and incubated 30 min at 37°C before being analyzed using a FACScan instrument (BD Bioscience), at the LCCC Flow Cytometry and Cell Sorting Shared Resource.

### Ethidium bromide staining

The possibility that Plumbagin treatment may induce apoptosis was evaluated by staining with ethidium homodimer (EthD-1), a cell viability indicator with high affinity for DNA that emits strong red fluorescence only in its DNA-bound state. EthD-1 is impermeant to healthy cells, but will stain cells undergoing apoptosis. HCT116 cells (8×10^4^) exposed to Plumbagin for 24 h as described above were stained with EthD-1 at 37°C for 1 h. The presence of red EthD-1 fluorescence was monitored by fluorescence microscopy.

### Detection of changes in mitochondrial transmembrane potential

The possible disruption of the mitochondrial potential in HCT116 cells by Plumbagin treatment was monitored using the MitoCapture™ Apoptosis Detection kit (BioVision, Milpitas, CA, USA) following the manufacturer’s instructions. This fluorescence-based assay detects the disruption or total loss of the mitochondrial transmembrane potential as one of the earliest intracellular events that occur following stimulation of apoptotic pathways. Cells were imaged immediately using an IX71 fluorescence microscope at the LCCC Microscopy and Imaging Shared Resource. Excitation was induced at either 478 or 507 nm, and emission (Em) was monitored with FITC (Em, 512–535) and rhodamine filters (Em, 540–560 nm).

### Western immunoblot analysis

Cells were lysed with RIPA buffer (50 mM Tris-HCl pH 7.4, 150 mM NaCl, 0.1% SDS, 1% deoxycholic acid, 1% NP-40, 1 mM EDTA) supplemented with a protease inhibitor cocktail (Roche Diagnostics, Indianapolis, IN, USA). Protein concentrations were determined using the BCA assay (Pierce/Thermo Fisher, Rockford, IL, USA) and a colorimetric plate reader. Extracts were electrophoresed on 4–20% gradient or 10% Tris-glycine SDS-polyacrylamide gels (Life Technologies, Grand Island, NY, USA), and the resolved polypeptides were transferred to polyvinylidene fluoride membranes. Transfers were performed at 25 V for 2 h at RT or 10 V overnight at 4°C. Non-specific binding was blocked by incubation with a solution of 5% skim milk in PBS containing 0.1% Tween-20 (PBS-T) at RT. After blocking, membranes were incubated with primary antibody and, following washes with PBS-T, were then incubated with horseradish peroxidase (HRP)-conjugated secondary antibodies, followed by visualization using the ECL (Pierce/Thermo Fisher) chemiluminescence detection system. Antibodies were prepared at the appropriate dilutions in blocking solution. For re-probing, membranes were incubated for 30 min at 50°C in stripping buffer (2% SDS, 62.5 mM Tris (pH 6.7) and 100 mM β-mercaptoethanol), rinsed thoroughly, and used again as described above.

### Measurement of reactive oxygen species

Intracellular ROS levels were determined using dihydrodichloro-fluorescein diacetate (H_2_DCF-DA), which is ultimately converted by oxidation into DCF, a fluorescent compound, in the presence of ROS. Cells (1×10^6^) treated with Plumbagin as described above were incubated at 37°C for 30 min with 10 μM H_2_DCF-DA, washed, resuspended in PBS, and immediately analyzed for fluorescence intensity with a fluorescence multi-well plate reader with excitation and emission wavelengths of 485 and 530 nm.

### Statistical analysis

Unless otherwise indicated, data are given as the mean ± standard error of the mean (SEM). All data were analyzed using a two-tailed paired Student’s t-test or one-way ANOVA, and values were considered to be statistically significant at p≤0.05.

## Results

Most cancer patients succumb to the consequences of metastatic cancer progression rather than as a result of their primary tumors (21). Cells dissociate from the primary solid tumors, enter the lymphatic and blood circulation, and disseminate through the body, ultimately homing in distant organs and forming colonies of metastatic cells (22). However, because normal cells and most tumor cells are known to die by anoikis when detached from an extracellular matrix (23,24), it is suggested that in the initial phases of their transient unattached state circulating tumor cells (CTCs) may in fact be stressed and, consequently, they might be more susceptible to the action of natural compounds with anticancer activity (25). In order to test any possible differential effect of Plumbagin on attached or unattached CRC cells, we utilized two treatment conditions: HCT116 cells were exposed to Plumbagin either i) at the seeding time, when they will remain unattached for several hours, or ii) 24 h after the cultures had been established, when all cells were already attached to the substrate.

### Plumbagin decreases the viability of CRC cells in a dose-dependent manner

**Figure 1:**
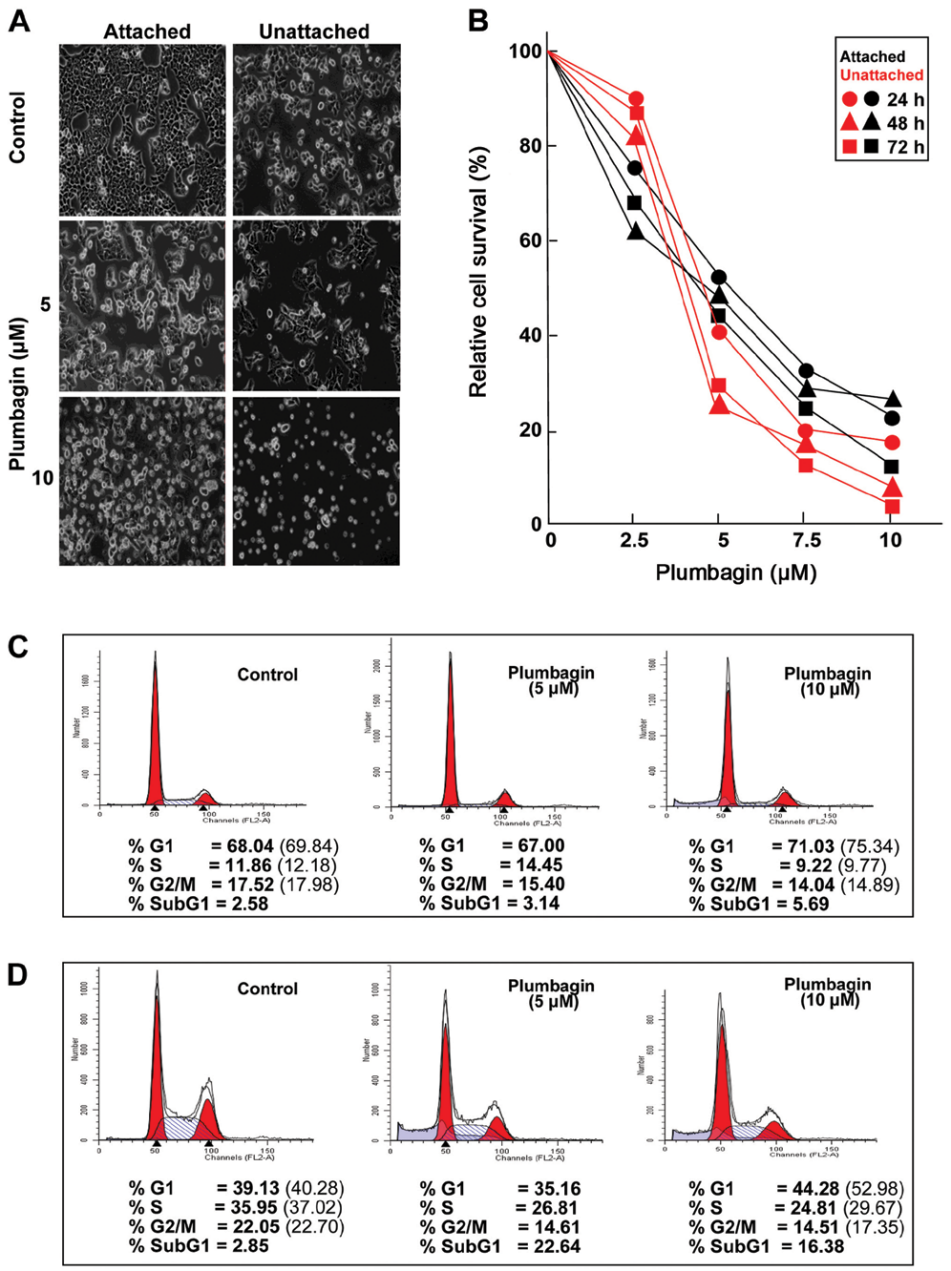
Effect of Plumbagin on cell proliferation. (A) Morphological changes observed on HCT116 cells after treatment for 24 h with the indicated doses of Plumbagin. The compound was added either when cells were already attached or at the time of seeding, when cells were still unattached for several hours; cells were observed by phase-contrast microscopy (x10). (B) Dose-dependent cytotoxicity effects of Plumbagin on HCT116 cells treated as indicated; results are expressed as the mean percentage of surviving cells relative to the untreated control cells; experiments were done in triplicate. Cell cycle analyses were performed with attached (C) and unattached (D) cells exposed for 24 h to the indicated doses of Plumbagin; cells were harvested, washed with PBS, fixed with ice cold 70% ethanol, stained with propidium iodide, and analyzed by flow cytometry; numbers in parentheses indicate relative percentages of cells in the indicated phases out of the total populations of cycling cells. In all cases, DMSO treated HCT116 cells served as control.

Regardless of the timing of addition, Plumbagin exposure had substantial effects on both attached and unattached HCT116 cells, which became obvious during morphological observation by phase-contrast microscopy (Fig. 1A) as well as in experiments designed to determine the viability of cultures treated with increasing drug concentrations (Fig. 1B). Plumbagin treatments for ≤72 h resulted in dose-dependent decreases in the viability of both attached and unattached cells, although unattached cells appeared to be somewhat more susceptible to the drug than the attached cells (Fig. 1B). On the basis of these results, two Plumbagin concentrations (5 and 10 μM) were chosen for further experiments. Cell cycle analyses were carried out to determine whether the observed effects on cell viability may be related to alterations in cell proliferation. The fact that Plumbagin exposure caused G1 arrest in cultures of either attached (Fig. 1C) or unattached (Fig. 1D) cells became apparent as early as 24 h after treatment initiation, even when the lowest (5 μM) concentration was used. Relative to vehicle-treated cultures, when considering only actively cycling cells, it became clear that exposure to 10 μM Plumbagin increased the percentage of attached cells (Fig. 1C) in G1 (7.9%), with concomitant decreases in the proportion of cells in the S (19.8%) and G2/M (17.2%) phases. The cell cycle effects of Plumbagin (10 μM) on unattached cells (Fig. 1D) were even more pronounced, showing a 31.5% increase in G1 cells, paralleled by decreases in the S (20.1%) and G2/M (23.6%) cell populations. In addition, comparisons of the relative proportions of cells detectable in the sub-G1 populations of attached (Fig. 1C) and unattached (Fig. 1D) cells in control and Plumbagin treated (10 μM) cultures revealed 2.24- and 6.19-fold increases, respectively, suggesting that cell death may also play a role in reducing the viability of HCT116 cells after drug exposure.

### Plumbagin treatment promotes apoptotic death of CRC cells

**Figure 2:**
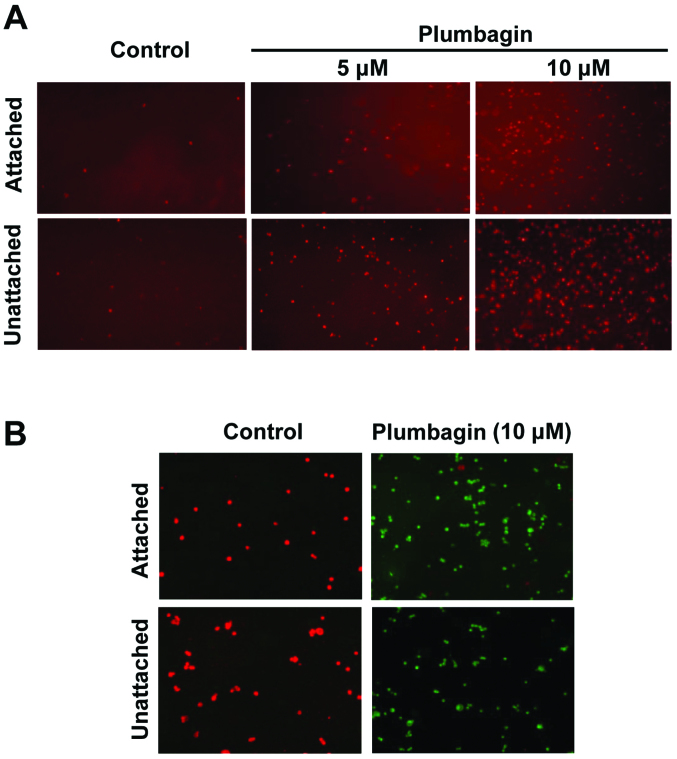
Plumbagin induced apoptosis of HCT116 cells via the mitochondria-mediated pathway. (A) Increased apoptosis were detected by fluorescence microscopy images (x100) of EthD-1 stained HCT116 cells after treatment for 24 h with the indicated doses of Plumbagin, added either when cells were already attached or at the time of seeding, when cells were still unattached. (B) Changes in mitochondrial transmembrane potential induced by Plumbagin in similarly treated cells were determined using the MitoCapture™ apoptosis detection kit. In all cases, DMSO treated HCT116 cells served as control.

To explore whether Plumbagin was causing the extent of cell death observed during cell cycle analyses (Fig. 1C and D) by promoting apoptosis, control and drug-treated HCT116 cells were subjected to ethidium bromide staining 24 h after treatment initiation, using the ethidium homodimer (EthD-1). This reagent is a cell impermeant viability indicator, which is a high-affinity nucleic acid stain that is weakly fluorescent until bound to DNA, when it emits intense red fluorescence. Results (Fig. 2A) showed a dose-related increase in fluorescence intensity that, in agreement with the relative proportion of sub-G1 populations after similar treatments (Fig. 1C and D), was clearly more pronounced in the case of Plumbagin-exposed unattached HCT116 cells. These findings demonstrated that Plumbagin exposure induced apoptosis of HCT116 cells, and that unattached cells were more susceptible to the drug treatment. To test the possible mitochondrial involvement in the apoptotic mechanism of Plumbagin action, control and drug-treated HCT116 cells were monitored using the MitoCapture™ system. In healthy cells, this cationic dye accumulates in mitochondria, yielding a bright red fluorescence, whereas in cells undergoing apoptosis mediated by alterations in the mitochondrial transmembrane potential it cannot aggregate in the mitochondria and remains in the cytoplasm giving off green fluorescence.

Results (Fig. 2B) showed that, while untreated cells consistently yielded red fluorescence only, almost no red-stained cells were detectable in cultures treated with 10 μM Plumbagin. The fact that the same pattern was observed in experiments using attached and unattached cells indicated that in both cases the apoptotic process triggered by Plumbagin exposure was mediated by alterations in the mitochondrial transmembrane potential induced by the drug.

### Plumbagin alters the expression of the cell cycle, apoptosis and proliferation markers in CRC cells

**Figure 3:**
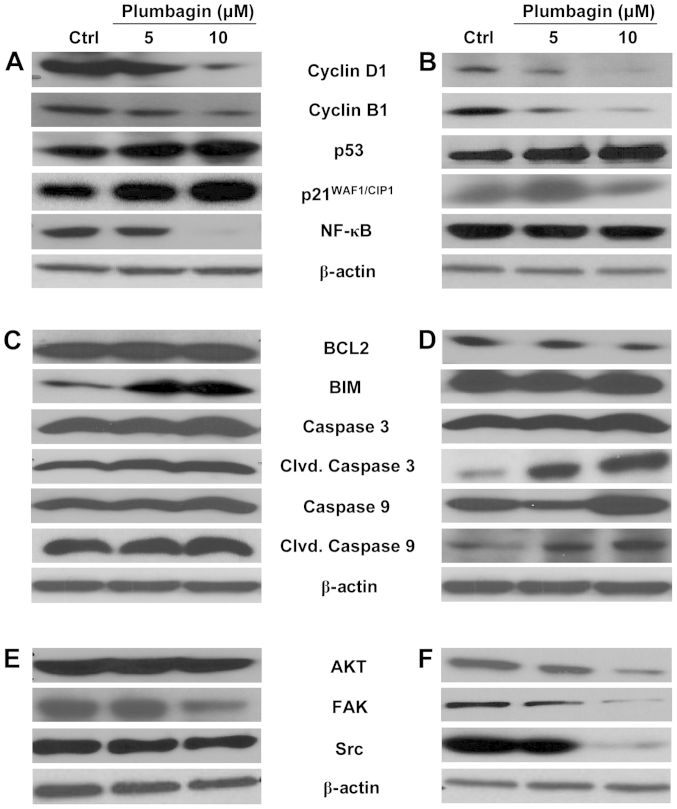
Expression analysis of cell cycle, apoptosis and proliferation marker proteins in Plumbagin treated HCT116 cells. Cells were treated for 24 h with the indicated doses of Plumbagin. The compound was added either when cells were already attached (A, C and E) or at the time of seeding, when cells remained still unattached for several hours (B, D and F). Cell extracts prepared after treatment were resolved by SDS-PAGE and analyzed by western immunoblot analysis with antibodies against the indicated proteins. Representative blots are shown for markers related to cell cycle (A and B), apoptosis (C and D) and cell proliferation (E and F). In all cases, DMSO treated HCT116 cells served as control (Ctrl). Human β-actin was used as the loading control.

Western immunoblot analyses were performed on total cell extracts prepared from control and Plumbagin-treated cells, to detect possible changes in the expression of specific gene products that could be associated with the anti-proliferative and apoptosis-inducing activity of the drug, and to identify possible differences between the action of Plumbagin on attached and unattached cells. As shown in Fig. 3, most Plumbagin-induced protein expression changes were similar between attached (Fig. 3A, C and E) and unattached (Fig. 3B, D and F) HCT116 cells, relative to their respective untreated controls. The differences observed were mainly related to the intensity of the stimulatory or inhibitory effects than to the nature of the protein products involved. With regard to cell cycle-related markers, Plumbagin decreased the expression of cyclin D1 and cyclin B1 and increased the expression of p53 and p21^WAF1/CIP1^ in a dose-dependent manner to similar extents in attached (Fig. 3A) and unattached (Fig. 3B) cells. However, the expression of NF-κB was almost completely abrogated in attached HCT116 cells exposed to 10 μM Plumbagin (Fig. 3A), whereas it was essentially unaffected by the same treatment in unattached cells (Fig. 3B). With regard to apoptosis-associated markers, as expected from the more pronounced pro-apoptotic activity of Plumbagin on unattached cells (Figs. 1 and 2), the expression of the anti-apoptotic protein BCL2 was reduced by drug treatment in the unattached cells, whereas it was essentially not modified by drug exposure in attached cultures. In addition, the increases in cleaved caspase 3 and cleaved caspase 9 caused by Plumbagin treatment were more pronounced in unattached cells (Fig. 3D) than in the attached cultures (Fig. 3C), although the total caspase 3 and caspase 9 contents were not altered in any case. It seemed rather interesting that the expression of the anti-apoptotic protein Bim was increased by drug treatment in attached cells (Fig. 3C), while it was essentially unchanged in unattached cells even after exposure to the highest (10 μM) Plumbagin concentration (Fig. 3D). The expression of other proliferation-associated proteins such as AKT, FAK and Src were markedly decreased by Plumbagin treatment in unattached HCT116 cells, in a dose-dependent manner (Fig. 3F), whereas unchanged AKT expression levels and only changes of lower magnitude in the expression of FAK and Src were detectable in the drug-treated, attached cells (Fig. 3E).

### Plumbagin induces the production of ROS by CRC cells

**Figure 4:**
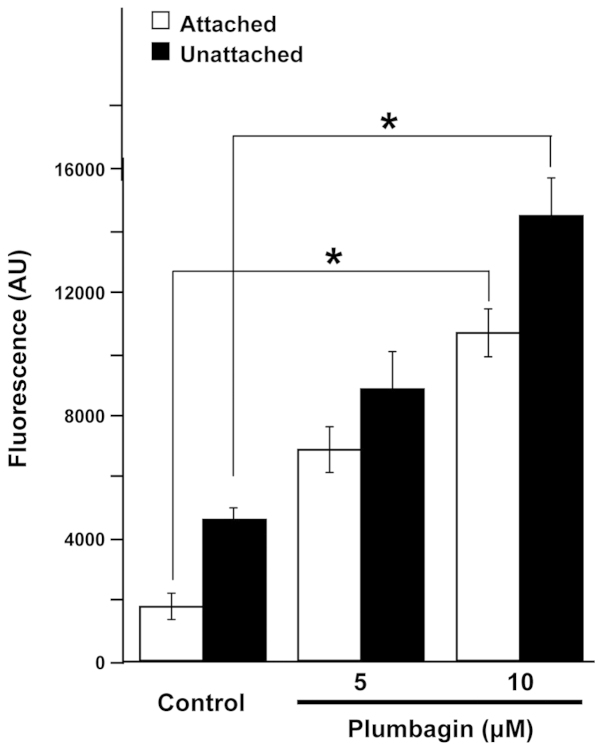
Generation of reactive oxygen species (ROS) in HCT116 cells after treatment with Plumbagin. Dose-dependent increase in ROS production by HCT116 cells after 24-h treatment with Plumbagin as described. ROS levels were determined using H_2_DCF-DA and detected fluorimetrically. Experiments were done in triplicate and results are expressed as the mean ± SD. ^*^Significance is indicated as p<0.01.

ROS have been suggested as possible triggers and/or effectors of apoptosis (26,27). To test whether the actions observed in CRC cells after Plumbagin exposure could be mediated by production of the superoxide anion, we determined the levels of intracellular ROS in control and Plumbagin-treated HCT116 cells using H_2_DCF-DA, a fluorescent dye which diffuses through cell membranes and in the presence of ROS is hydrolyzed by intracellular esterases to DCF, which is highly fluorescent. Results (Fig. 4) showed that Plumbagin exposure increased the intracellular levels of ROS in both attached and unattached HCT116 cells, in a dose-dependent manner, reaching levels of production that were statistically significant (p<0.01) in both experimental scenarios when treatments were carried out by exposing the cells to 10 μM Plumbagin.

## Discussion

Natural products are considered as one of the most important sources of promising leads for the development of novel anticancer chemotherapeutics. Of special interest are those of plant origin, the so-called ‘nutraceuticals’ (28), that by being part of the human diet have already demonstrated low toxicity levels. Studies of these compounds, particularly those that may have the ability to stimulate tumor cell apoptosis (4,29) may lead to the discovery of new anticancer drugs among the large pool of plant secondary metabolites, which provide a great variety of chemical structures (30). Our studies have focused on the effects of Plumbagin, a quinone derived from plant secondary metabolites, on colon cancer cells that derive from the tissue type most likely to be the immediate target of Plumbagin and its metabolites after ingestion (31).

Although the potential anticancer activity of Plumbagin has been tested on various human tumor types (32–37), and despite the fact that studies in a rat model for azoxymethane-induced intestinal carcinogenesis identified Plumbagin as a promising chemopreventive agent (13), few studies on its effect on human colon cancer have been reported (38,39). Our results clearly demonstrate that Plumbagin has a potent action against HCT116 colon cancer cells regardless of whether attached or unattached cultures were exposed to the drug. In general, the overall survival effects and G1 cell cycle arrest were quite similar between attached and unattached cells (Fig. 1B–D), although there were also some obvious differences. The extent of cell death caused by Plumbagin on unattached cells was 3–4-fold higher than in drug-treated attached cultures, as reflected by the proportion of sub-G1 cells detectable in each case (Fig. 1C and D). Nevertheless, the observed cell cycle and apoptosis effects of Plumbagin were consistent with the changes detected, at the protein level, in the expression of genes relevant to cell cycle control, cell death response and the regulation of cell proliferation (Fig. 3). In general, the nature of the effects of Plumbagin on protein expression detected in our case agreed with those reported earlier on other colon cancer cell lines (38,39). However, because we used lower concentrations of Plumbagin (≤10 μM), compared with the concentrations used in those studies (≤75 μM), it seems that HCT116 cells are more sensitive than HT-29 and HCT15 cells. In addition, within our own experimental system there was a high level of coherence among the various results obtained: i) the cell cycle arrest observed was consistent with the decreased levels of cyclins D1 and B1, together with the increased levels of p53 and p21^WAF1/CIP1^; ii) the induction of apoptosis through the involvement of mitochondrial pathways suggested by fluorescence microscopy after specific cellular stains (Fig. 2) was consistent with increased levels of cleaved-caspase 3 and cleaved-caspase 9; and iii) the decreased expression of proliferation marker proteins was also consistent with the global decrease in the size of the proliferating cell population.

In general, the magnitude of the observed effects was the main difference between the action of Plumbagin on attached and unattached cells. However, it appears that there is a clear difference in the mechanism by which the drug induces apoptosis under the different experimental conditions: unaltered BCL2 expression (40) and increased BIM expression (41) in the attached cells, in contrast to decreased BCL2 and unaltered BIM expression in the unattached cells. The fact that these differences were observed with both Plumbagin concentrations used suggests that another apoptosis-regulatory protein may be differentially affected by the drug and contribute to the ultimate decision between the two pro-apoptotic mechanisms.

Another important point of contrast, not only between attached and unattached cells in our system, but also in relation to the published literature on the anticancer action of Plumbagin is the effect on NF-κB expression. Some reports indicated that Plumbagin treatment increased NF-κB (38), whereas others described not only that NF-κB was decreased by drug treatment (36,42), but even that Plumbagin exposure suppressed the NF-κB increasing effect of ionizing radiation (43). In our experimental system, NF-κB remained essentially unchanged by Plumbagin treatment in unattached cells (Fig. 3B), whereas it was reduced to nearly undetectable levels by drug treatment in attached cells (Fig. 3A). At present, there is no obvious explanation for this discrepancy. Additional experiment will be required to answer this question. What seems to be universally consistent among data reported in the literature (14,44) as well as in our own system (Fig. 4) is the ability of Plumbagin to promote the production of ROS, which agree with its known pro-oxidant nature (45). The levels of ROS production by Plumbagin-treated cells agree well with the overall extent of survival inhibition caused by the drug under attached and unattached culture conditions.

Overall, our data indicate that Plumbagin treatment had a dual effect on HCT116 cells: the induction of ROS formation, which promoted apoptosis via the mitochondrial cell death pathway, and the simultaneous induction of cell cycle arrest at the G1 phase with associated increases in the levels of p53 and p21^WAF1/CIP1^. The fact that these effects were observed both in attached cells as well as in cells maintained under unattached conditions strongly suggests that Plumbagin treatment may be effective not only on cells in primary solid tumors but also on cells that may have dissociated from the primary tumors and are transiently suspended in biological fluids on their way to homing in distant tissues where they will establish metastatic colonies. Therefore, it seems evident that Plumbagin is a promising anticancer drug with potential therapeutic uses for the treatment of CRC patients.
